# Dicer Is Required for Maintaining Adult Pancreas

**DOI:** 10.1371/journal.pone.0004212

**Published:** 2009-01-16

**Authors:** Sumiyo Morita, Akemi Hara, Itaru Kojima, Takuro Horii, Mika Kimura, Tadahiro Kitamura, Takahiro Ochiya, Katsumi Nakanishi, Ryo Matoba, Kenichi Matsubara, Izuho Hatada

**Affiliations:** 1 Laboratory of Genome Science, Biosignal Genome Resource Center, Institute for Molecular and Cellular Regulation, Gunma University, Showa-machi Maebashi, Japan; 2 Japan Health Sciences Foundation, Chuo, Tokyo, Japan; 3 Department of Molecular Medicine, Institute for Molecular and Cellular Regulation, Gunma University, Showa-machi Maebashi, Japan; 4 Metabolic Signal Research Center Laboratory of Metabolic Signal, Institute for Molecular and Cellular Regulation, Gunma University, Showa-machi Maebashi, Japan; 5 National Cancer Center Research Institute, Section for Studies on Metastasis, Tsukiji, Chuo-ku, Tokyo, Japan; 6 DNA Chip Research Inc., Suehirocho, Tsurumi-ku, Yokohama, Japan; University of Michigan, United States of America

## Abstract

*Dicer1*, an essential component of RNA interference and the microRNA pathway, has many important roles in the morphogenesis of developing tissues. *Dicer1* null mice have been reported to die at E7.5; therefore it is impossible to study its function in adult tissues. We previously reported that *Dicer1*-hypomorphic mice, whose *Dicer1* expression was reduced to 20% in all tissues, were unexpectedly viable. Here we analyzed these mice to ascertain whether the down-regulation of *Dicer1* expression has any influence on adult tissues. Interestingly, all tissues of adult (8–10 week old) *Dicer1*-hypomorphic mice were histologically normal except for the pancreas, whose development was normal at the fetal and neonatal stages; however, morphologic abnormalities in *Dicer1*-hypomorphic mice were detected after 4 weeks of age. This suggested that *Dicer1* is important for maintaining the adult pancreas.

## Introduction

MicroRNA (miRNA) is small (∼22 nucleotides), non-coding RNA. Mature miRNA transcribed as long primary transcripts is processed to pre-miRNA in the nucleus by *Drosha*/*DGC8* [1], and then processed in the cytoplasm by *Dicer* [2]. MiRNA is further incorporated into the RNA-inducing silencing complex (RISC), which includes Argonaute [3] to regulate gene expression via post-transcriptional repression. Over the past few years, more than 400 miRNAs have been identified, but their function is largely unknown. Several miRNAs exhibit tissue-specific or developmental stage-specific expression [4], [5], indicating that they have important roles in many biological processes.

*Dicer1* encodes an RNaseIII endonuclease, a key enzyme that processes miRNA. It is broadly expressed in developing tissues, and several mutant alleles of *Dicer1* have been generated in mice. *Dicer1* seems to be critical in early development since loss of its function was lethal at embryonic day 7.5 [6]. Characterization of *Dicer1* hypomorphic mice showed that the gene is required for embryonic angiogenesis [7]. Conditional inactivation of *Dicer1* in the mouse limb bud mesenchyme [8], lung epithelium [9], epidermal hair follicle [10], and pancreas [11], T cell development and differentiation [12] led to the conclusion that *Dicer1*, which processes miRNA, is indispensable for the development and morphogenesis of these tissues.

We previously generated *Dicer1*-hypomorphic mice (homozygous *Dicer1*−/− mice) [13]. Complete loss of *Dicer1* in mice results in early embryonic death [6]; however, our *Dicer1*-hypomorphic mice were viable [13]. To study the function of *Dicer1* in the maintenance of homeostasis in adult tissues, we analyzed the adult tissues histologically and found abnormalities only in the pancreas. The phenotypes detected in the pancreas of *Dicer1*-hypomorphic mice might resemble the differentiation of endocrine precursor cells in adult pancreas.

The pancreas consists of three main tissue cell types: the endocrine cells (islet of Langerhans) which produce hormones such as insulin and glucagon; the exocrine acinar tissues which secrete digestive enzymes; and the branched duct. Numerous mechanisms that control the differentiation of endocrine and exocrine cells in the embryonic pancreas have been revealed [14], but how endocrine cells (especially insulin-producing β cells) are maintained in postnatal life has been controversial [15]. At E9.5, the endocrine cells of the pancreas arise from endocrine precursor cells, which express both glucagon and insulin and divide into distinct lineages such as glucagon or insulin-expressing cells. On the other hand, in the adult pancreas, it had been considered that there are no endocrine progenitor cells and that β cells are generated only by the replication of existing β cells, not from the differentiation of endocrine precursor cells (neogenesis) [16], [17]. However, several studies suggested that β cell differentiation from endocrine precursor cells can occur in adults in the regenerating pancreas after a partial pancreatectomy or duct ligation [18], [19], [20], [21]. In the regenerating pancreas, vigorous expansion of the β cell population was observed, and partial pancreatectomy and duct ligation has been a good model for regenerating endocrine cells. The phenotypes observed in *Dicer1*-hypomorphic mice suggested that *Dicer1* regulates the endocrinal neogenesis in the adult pancreas. Previous study showed that *Dicer1* is indispensable for normal development of the pancreas [11]; however, its function in the adult pancreas had not been elucidated. Here we report that *Dicer1* also has important functions in the adult pancreas.

## Results

### *Dicer1* expression was significantly reduced in all tissues of *Dicer1*-hypomorphic mice but histological abnormalities were only found in the pancreas

**Figure 1:**
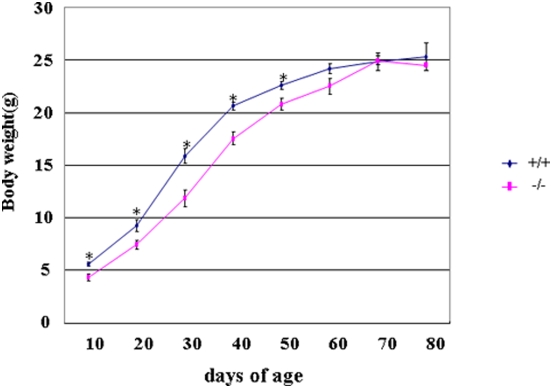
Body weight growth curves. Male wild-type (+/+) and *Dicer1*-hypomorphic (−/−) mice were measured to determine the change in body weight from 10 to 80 days of age. *, P<0.05. n = 8–10 per group.

**Figure 2:**
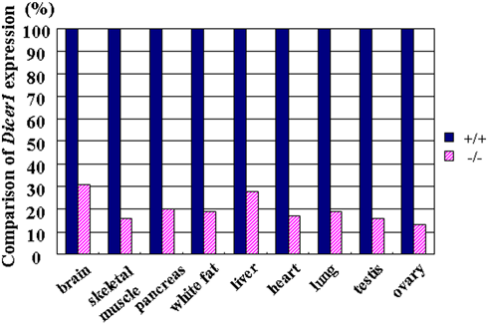
Comparison of *Dicer1* expression in nine tissues between wild-type (+/+) and *Dicer1*-hypomorphic (−/−) mice. The expression in the *Dicer1*-hypomorphic mice was normalized to that in the wild-type mice.

**Figure 3:**
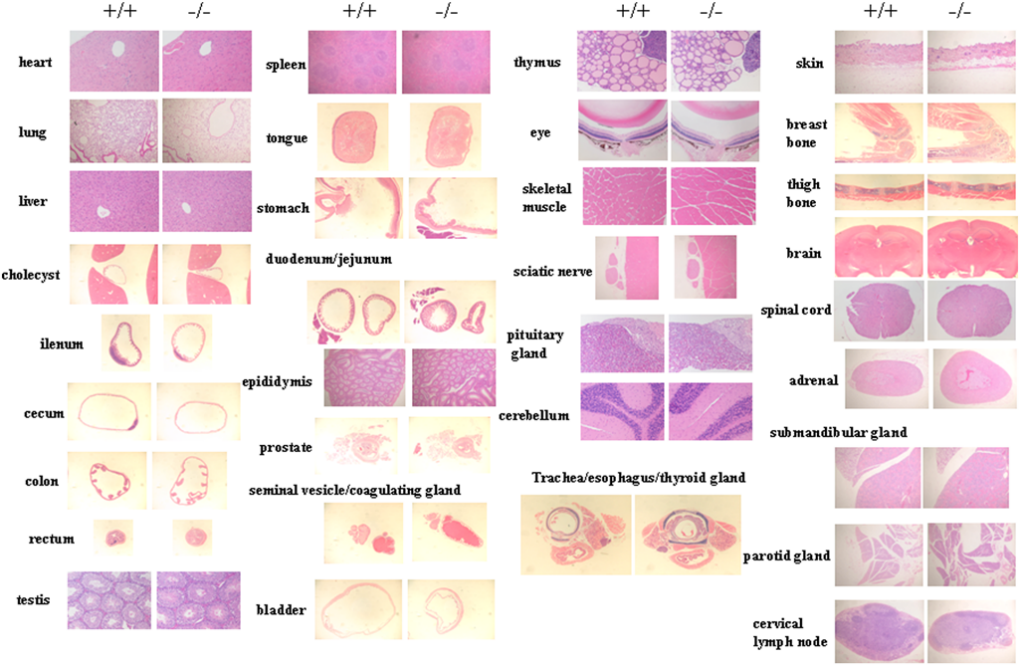
H&E-stained section of adult tissues of wild-type (+/+) (n = 2) and *Dicer1*-hypomorphic (−/−) mice (n = 4).

*Dicer1*-hypomophic mice (homozygous *Dicer1*−/− mice) showed a lower birth rate than expected by Mendelian rules [13]; however, they did not differ from their wild-type littermates in overall health. Although they showed slight growth retardation from 10 to 50 days of age, their body weight was similar to that of wild-type mice after 50 days of age (Fig. 1). A comparison of *Dicer1* expression in nine tissues of adult mice revealed a 70–85% reduction in the hypomorphic mice (Fig. 2). Although we analyzed more than 40 tissues (Table 1), histological examination revealed no abnormalities in any tissues except the pancreas (Fig. 3); thus we focused on the pancreas of *Dicer1*-hypomorphic mice.

**Table 1:** The list of tissues with histological analysis (H&E assessment).

lung	testis	eye boll	trachea
heart	epididymis	harderian gland	esophagus
kidney	prostate	skeletal muscle	thyroid gland
pancreas	seminal vesicle	sciatic nerve	liver
tongue	coagulating gland	skin	cholecyst
stomach	bladder	breast bone	spleen
duodenum	adrenal	femur	
jejunum	pituitary gland	cerebrum	
ileum	submandibular gland	hippocampus	
cecum	parotid gland	thalamus	
colon	thymus	cerebellum	
rectum	cervical lymph node	spinal cord	

Histological analysis of these tissues was performed in wild-type (+/+) (n = 2) and *Dicer1*-hypomorphic (−/−) mice (n = 4).

### *Dicer1* could be involved in differentiation of endocrine cells in adult pancreas

**Figure 4:**
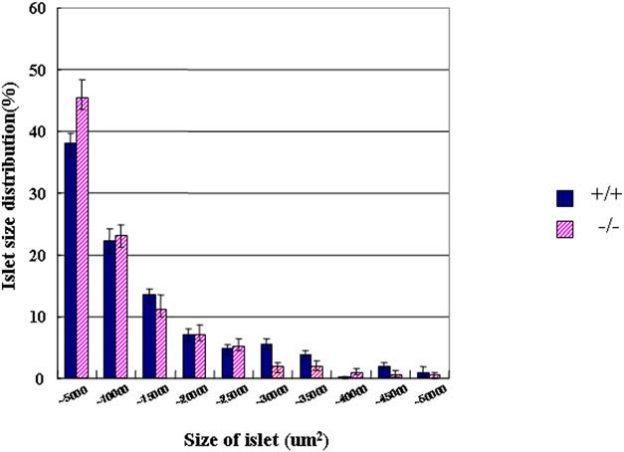
Comparison of the size of islets in wild-type and *Dicer1*-hypomorphic mice. The islets mass was measured in wild-type (blue bar) and *Dicer1*-hypomorphic mice (pink bar). The numbers of islets were examined in wild-type (n = 6) and *Dicer1*-hypomorphic (n = 6) mice, with six sections from each animal. The graph shows the percentage of islets in each size category.

**Figure 5:**
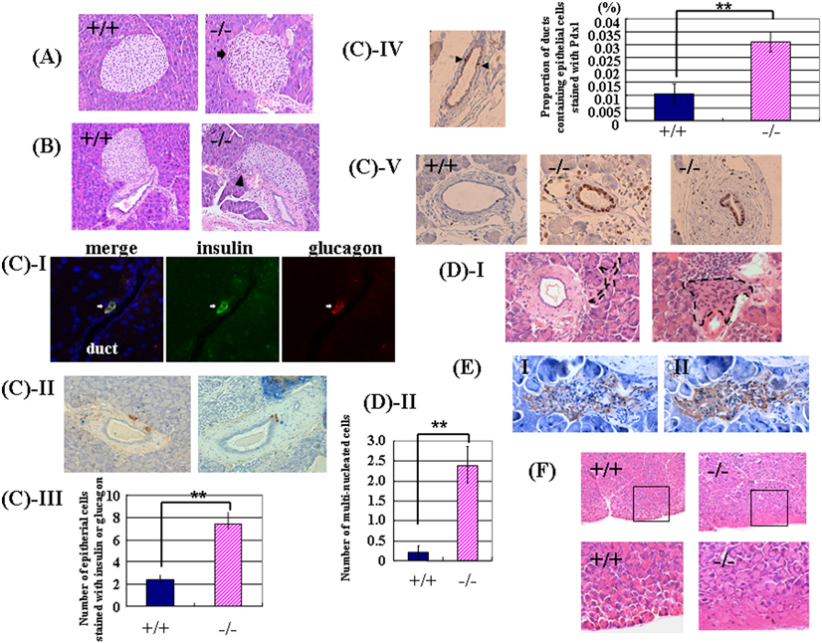
Pancreas morphology in adult (8–10 weeks of age) *Dicer1*-hypomorphic mice. A, B: Hematoxylin-eosin (H & E)-stained islets of the pancreas from an 8-week-old wild-type (+/+) mouse and *Dicer1*-hypomorphic (−/−) mice (*400). A: Arrows indicate an irregular distribution of islet cells. B: Arrowheads indicate that the boundary of islets and ducts was not clearly defined in the pancreas of *Dicer1*-hypomorphic mice. C: Immunohistochemistry of duct cells of *Dicer1*-hypomorphic mice. (I) Insulin (green) and glucagon (red) double-expressing cells were detected in the duct. (II) Insulin-positive cells (brown) and glucagon-positive cells (blue) were observed in the duct. (III) Comparison of the number of epithelial cells stained with both insulin and glucagon, only insulin, and only glucagon in wild-type and *Dicer1*-hypomorphic mice. These numbers were averaged from 6 animals, with six sections from each animal. **, P<0.01. (IV) Comparison of the proportion of ducts containing epithelial cells stained with Pdx1 in wild-type and *Dicer1*-hypomorphic mice. Arrowheads indicate the Pdx1-positive cells. *, P<0.01. (V) Abnormal staining of Ki67 was observed in the pancreas of *Dicer1*-hypomorphic mice. D: (I) H & E-stained multinuclear atypical cells in the pancreas of *Dicer1*-hypomorphic mice. The black dotted line indicates atypical multinuclear cells. (II) Comparison of the number of multi-nucleated cells in wild-type and *Dicer1*-hypomorphic mice. These numbers were averaged from 6 animals, with six sections from each animal. **, P<0.01. E: Immunohistochemistry of multinuclear atypical cells of adjacent sections of the pancreas of *Dicer1*-hypomorphic mice using anti-insulin (I) and anti-glucagon (II) antibodies. F: H & E-stained acinar cells. The rectangular areas outlined in the upper panels are magnified in the lower panels. An abnormal structure of exocrine cells was observed in the pancreas of *Dicer1*-hypomorphic mice.

In *Dicer1*-hypomorphic mice, the size of the pancreas in adults (8–10 weeks of age) was nearly identical to that in the wild-type mice; however, there were more small islets (Fig. 4). In some of these islets, the distribution of islet cells and staining of nuclei were irregular (Fig. 5A). The boundary of islets and ducts was not clearly defined in the pancreas (Fig. 5B). Immunohistochemical analysis revealed mostly normal staining of insulin and glucagon at 8–10 weeks of age; however, the number of ductal epithelial cells stained with insulin or glucagon was significantly increased (Fig. 5C-III, P = 0.0051). In some models of pancreatic regeneration including partial pancreatectomy, insulin or glucagon-stained cells are present in the ductal epithelium, which had led to the idea that some endocrine cells differentiate in the ducts [18], [19], [20], [21]. Our observations in *Dicer1*-hypomorphic mice suggest that regeneration from the endocrine precursor cells took place in adulthood. Next we conducted a histological examination of the markers Pdx-1 and Ki67. The population of ducts containing Pdx-1-positive cells was significantly increased (Fig. 5C-IV, P = 0.009). Pdx1-positive cells in the ducts are possibly the adult progenitor cells [22], [23], and PDX-1 protein was detected in the pancreatic duct in adult rats after partial pancreatechtomy [21]. Surprisingly, abnormal staining of Ki67, which is a marker for proliferation of the cells, was detected in the pancreatic ducts in two of six *Dicer1*-hypomorphic mice (Fig. 5C-V). In some Ki-67-positive ducts, all the epithelial cells were stained. No such observations were found in wild-type mice.

Interestingly, cells morphologically different from either acinar or islet cells were observed in *Dicer1*-hypomorphic mice (Fig. 5D-I). Under a light microscope, some appeared to be syncytial multi-nucleated cells near the pancreatic duct and in acini. Numerous nuclei were distributed irregularly and were often clustered in the cells, which were all double-positive for insulin and glucagon (Fig. 5E). Cells double-positive for insulin and glucagon were significantly increased in *Dicer1*-hypomorphic mice compared to wild-type mice (Fig. 5D-II, P = 0.0019). In the exocrine portion of the pancreas of *Dicer1*-hypomorphic mice, most acini were morphologically normal, but some showed an irregular morphology (Fig. 5F). The shapes and position of the cells were irregular and the acinar structure was not organized. In normal acinar cells, zymogen granules are observed in the center of the acinus and the nucleus is located at its periphery.

**Figure 6:**
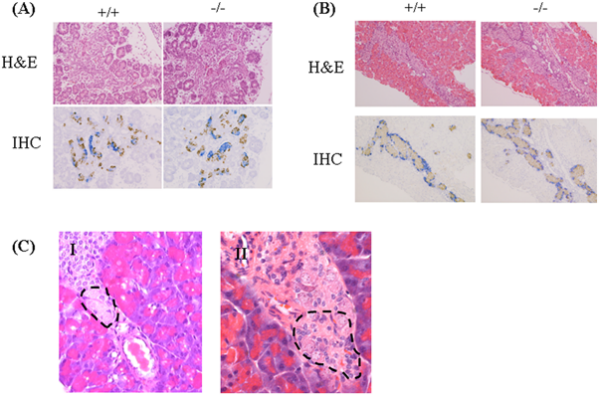
Histological and immunohistochemical analysis of the pancreas at E15.5 (A) and P1 (B) of wild-type and *Dicer1*-hypomorphic mice. *Dicer1*-hypomorphic mice show normal insulin (brown) and glucagon (blue) staining at E15.5 and P1. C: Histological abnormalities found in the pancreas of 4-week-old *Dicer1*-hypomorphic mice. (I) The endocrinal distribution was slightly irregular. The dotted line indicates the abnormal region of the islet. (II) Multi-nucleated cells were observed. The dotted line indicates multi-nucleated cells, which were also found in the pancreas of adult *Dicer1*-hypomorphic mice.

We next investigated when the abnormal morphology appeared in the development of the pancreas in *Dicer1*-hypomorphic mice. For this purpose, a histological analysis was performed using E15.5 embryos, P1 mice, and 4-week-old mice. The pancreas of both wild-type and *Dicer1*-hypomorphic mice developed normally and endocrine and exocrine cells appeared morphologically normal at E15.5 and P1 (Fig. 6A, B); the same abnormalities observed in adult *Dicer1*-hypomorphic mice were detectable at 4 weeks of ages (Fig. 6C), although the number of abnormal cells was less than that found in adult *Dicer1*-hypomorphic mice. This suggested that the pancreas of *Dicer1*-hypomorphic mice developed normally after birth and abnormal cells appeared at around 4 weeks after birth, increasing with age.

**Figure 7:**
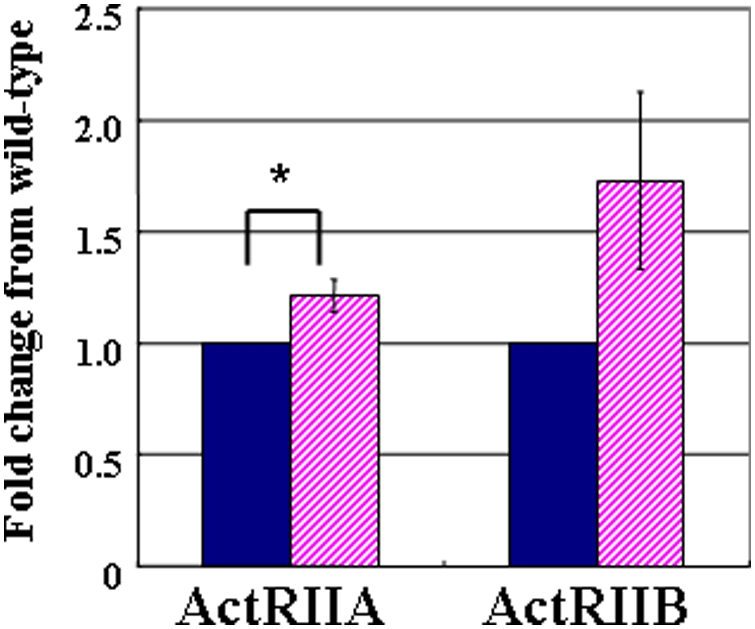
Analysis of ActRIIA and ActRIIB expression. Data are expressed relative (n-fold) to the wild-type pancreas and correspond to the means and standard errors for three independent experiments performed in triplicate. *, P<0.05. wild-type n = 9, *Dicer1*-hypomorphic mice n = 9.

Surprisingly, the observations found in *Dicer1*-hypomorphic mice were quite similar to the histological findings in transgenic mice expressing a truncated type II activin receptor [24]. Therefore, we next investigated the expression of the activin type II receptor in the pancreas of *Dicer1*-hypomorphic mice. Two related receptors, ActRIIA and ActRIIB, were initially identified as type II receptors for activin [25], [26]. ActRIIA and ActRIIB have been reported to bind not only to activin [27], but also to other TGF-β family proteins, including BMP7 [28], GDF8 [29], Nodal [30], and GDF11 [31]. The precise role of the two activin receptors is still not clear. Real-time PCR analysis revealed that the expression of ActRIIA was slightly up-regulated in *Dicer1*-hypomorphic mice compared to wild-type mice, while the expression of ActRIIB did not differ (Fig. 7). Therefore, the abnormal morphology might be attributed to another signaling cascade.

### Detection of differential expressed miRNAs by microarray analysis

**Figure 8:**
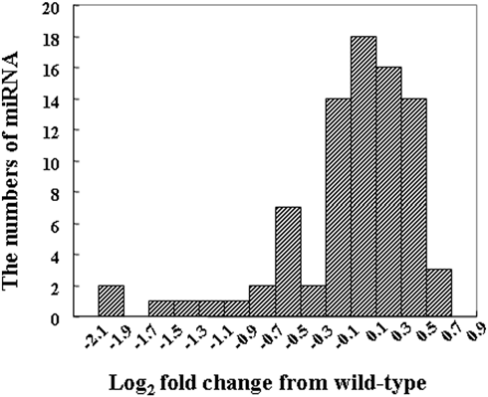
The distribution of changes in miRNA levels in *Dicer1*-hypomorphic mice compared to wild-type mice.

Because *Dicer1* is required for the processing of miRNAs, the reduction of *Dicer1* results in a decrease in miRNAs. To determine the differential expression of miRNAs in the pancreas of adult wild-type and *Dicer1*-hypomorphic mice, a miRNA microarray analysis was performed. The miRNAs of the pancreas of two wild-type and two *Dicer1*-hypomorphic mice were analyzed. Signals were very weak on hybridization with miRNA in the pancreas compared to other tissues; therefore a total of 83 miRNAs, which showed strong signals, were analyzed. Fig. 8 shows the change in the distribution of miRNA levels in *Dicer1*-hypomorphic mice compared to wild-type mice. Surprisingly, miRNA expression did not dramatically change in *Dicer1*-hypomorphic mice compared to the wild-type animals: however, 7% of miRNAs were down-regulated less than 0.5 fold. These miRNAs might function in maintaining the adult pancreas, but at present their relationship with the abnormal phenotype in *Dicer1*-hypomorphic mice is unclear. Why was only 7% of the miRNA expressed in pancreas attenuated? Dicer1 protein might catalyze processing of pre-miRNA differently dependent on the sequence when generating miRNA. The down-regulated miRNAs might be more difficult to process than the other miRNAs and thus significantly reduced *Dicer1* expression might affect their generation.

### Glucose metabolism was normal in *Dicer1*-hypomorphic mice

**Figure 9:**
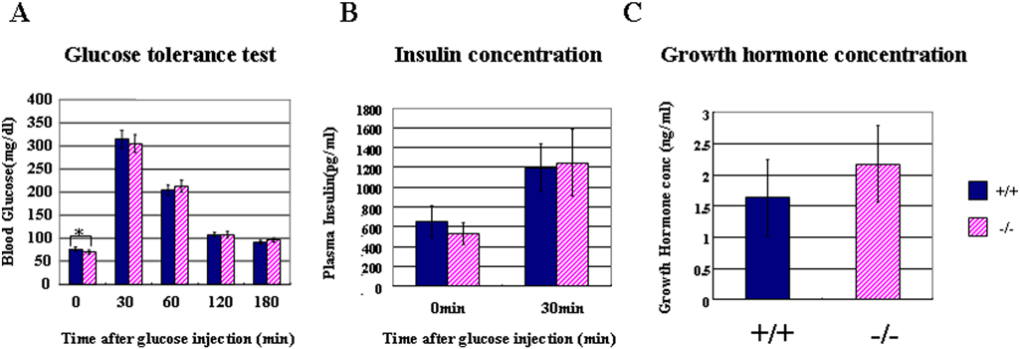
Glucose metabolism and growth hormone levels in wild-type and *Dicer1*-hypomorphic mice. A: Glucose tolerance test. Fasted 8-week-old mice received an intraperitoneal injection of glucose (2 mg/g body weight). B: Insulin concentration. Plasma insulin was measured before and after the intraperitoneal glucose injection. C: Growth hormone concentration. Plasma growth hormone concentrations were measured in wild-type and *Dicer1*-hypomorphic mice. A, B: Values are expressed as the means±S.D. (n = 20 per group). C: Values are expressed as the means±S.D. (n = 10 per group).*, P<0.05.

The hypo-expression of *Dicer1* leads to abnormal endocrine cells, which might affect the function of the pancreas; therefore, we next investigated the metabolism of glucose in *Dicer1*-hypomorphic mice. Despite histological abnormalities in pancreatic islets, a glucose tolerance test showed that *Dicer1*-hypomorphic mice were able to clear glucose from the blood as efficiently as wild-type mice (Fig. 9A), and had insulin levels similar to wild-type mice (Fig. 9B). Despite no significant differences in the insulin content of serum after overnight fasting, *Dicer1*-hypomorphic mice showed a slightly reduced blood glucose level on fasting. *Dicer1*-hypomorphic mice were smaller than the wild-type mice before 50 days of age; therefore, we hypothesized that the growth hormone level affects fasting hypoglycemia. We checked the blood growth hormone level; however, we found no significant differences with wild-type mice (Fig. 9C). Thus, the growth hormone level is not responsible for fasting hypoglycemia in *Dicer1*-hypomorphic mice, probably due to an unknown mechanism involved in glucose metabolism in other tissues.

## Discussion

*Dicer*, the enzyme that generates miRNAs, has been reported to have quite important roles in a variety of developmental processes. In our *Dicer1*-hypomorphic mice, histological analysis (H&E assessment) showed that the abnormalities were found only in the pancreas and not in other tissues. However, we could not exclude the possibility that there are more minute structural abnormalities not detected with the H&E assessment, or functional abnormlities. In this study, we focused on the pancreas. The pancreatic-specific knockout of *Dicer1* clarified that miRNA is required for the development of the pancreas in embryogenesis [11]. Our study suggested that *Dicer1* also has important functions in maintaining the adult pancreas.

Histological abnormalities such as the irregular distribution of islet cells, and deviations from the typical structure of the acinus, were found in endocrine and exocrine cells in the adult pancreas of *Dicer1*-hypomorphic mice, although none of these abnormalities were detected before 4 weeks of age. *Dicer1* is indispensable for normal pancreatic cell differentiation at embryogenesis [11]. The pancreas-specific *Dicer1* knockout mice survived until birth but died before P3. These mice showed gross defects in all pancreatic lineages, and the formation of exocrine cells and duct cells, especially endocrine cells, was greatly impaired. Given that miRNA most probably plays essential roles in the morphogenesis of many tissues in a developing embryo [7], [8], [9], [10], [12], the gross defects in all pancreatic lineages observed upon removal of *Dicer1* are not surprising. These mice died soon after birth; therefore, it is impossible to study the role *Dicer* may play in adult tissues.

The pancreas of our *Dicer1*-hypomorphic mice developed normally and the reduced expression of *Dicer1* did not affect the development of the pancreas during embryogenesis or the neonatal stage. However, aberrant endocrine and exocrine cells could be detected after 4 weeks of age, and the number of abnormal regions seemed to increase with age. It is interesting that the developing pancreas during embryogenesis and the adult pancreas differ in sensitivity to the *Dicer1* level. In other words, the reduction in *Dicer1* only affects the maintaining of adult pancreas, not the normal development of the pancreas.

In addition, these observations, such as the increasing number of ductal epithelial cells stained positive for insulin, glucagon, and Pdx-1 in *Dicer1*-hypomorphic mice (Fig. 5D-III, IV), were also found in the regenerating pancreas [18], [19], [20], [21], suggesting that the differentiation of endocrine precursor cells (neogenesis) occurred in the adult pancreas. Moreover, a quite intriguing observation was the existence of unknown abnormal multi-nucleated cells in the pancreas that expressed both glucagon and insulin. In the developing pancreas, endocrine precursor cells first appeared at E9, and these cells expressed both glucagon and insulin [32], [33], then differentiated into insulin-producing cells or glucagon-producing cells. These cells in our adult *Dicer1*-hypomorphic mice resembled endocrine precursor cells in the fetal pancreas in terms of the expression of both glucagon and insulin. Therefore, *Dicer1* might have roles in regulating endocrine precursor cells in the adult pancreas.

The proliferation of duct cells is increased in the regenerating pancreas compared to the normal adult pancreas [20]. However, in some *Dicer1*-hypomorphic mice, abnormal proliferation of duct epithelial cells was observed (Fig. 5D-V). These features were not detected in wild-type mice and could be caused by the reduction in *Dicer1*.

Surprisingly, these histological observations in *Dicer1*-hypomorphic mice were quite similar to the histological findings in transgenic mice expressing the truncated type II activin receptor [24]. However, as our *Dicer1*-hypomorphic mice showed only a 1.2-fold increase in ActRIIA in the pancreas, ActRIIA might not cause the abnormal phenotype.

Because *Dicer1* has a key role in generating a large number of miRNAs, its removal results in a significant decrease in miRNAs. In the pancreas of our *Dicer1*-hypomorphic mice, the expression levels of miRNA changed slightly compared to wild-type level which is why the mice could survive. A complete loss of *Dicer* in mice results in early embryonic death. A large number of genes control the development or maintenance of the pancreas, and these genes might be a potential target of miRNAs. Even a slight change in miRNA expression might affect the gene expression, leading to the abnormal morphology in *Dicer1*-hypomorphic mice. Further exploration is necessary to investigate the relation between these genes and miRNA in the pancreas.

Our results suggest that *Dicer1* functions in the adult pancreas and also raise the possibility that *Dicer1* regulates the differentiation of endocrine precursor cells there. Further analysis is necessary to understand the mechanism behind the maintenance of each cell type in the adult pancreas, especially β cells.

## Materials and Methods

### Gene targeting and mice

We generated *Dicer1*-deficient mice from an ES cell (RFF266), which was obtained from Bay Genomics [34], carrying a gene trap insertion between exon 22 and exon 23, resulting in disruption of the second RNaseIII domain and loss of the double-stranded RNA-binding domain. A gene trap vector called pGT1Lxf, which has a splicing acceptor, was used to make this ES cell. Targeted clones were injected into blastocysts to generate chimeras. Five chimeras were generated and backcrossed with C57BL/6 mice. *Dicer1* heterozygous mice were backcrossed with C57BL/6 mice for 12 generations. All animal experiments were approved by the Animal Research Ethics Board at the Gunma University.

### Histochemistry and immunohistochemistry

Tissues and embryos were fixed overnight in formalin at 4°C and embedded in paraffin. Standard techniques were used for the embedding, sectioning and staining of tissues. Sections were cut at 5 µm. Immunohistochemistry was performed as follows: The slides were dewaxed and washed in PBS, and blocked in 1% BSA for 30 min. They were then incubated with primary antibodies overnight at 4°C in PBS containing 1% BSA, washed in PBS, and incubated with the appropriate secondary antibodies for 1 hour at room temperature. The slides were washed in PBS and mounted with Pristine Mount (Parma) with DAPI. The primary antibodies used were rabbit anti-glucagon (1∶200, DAKO) and guinea pig anti-insulin (1∶200, DAKO), rabbit Ki67 monoclonal antibody (Lab Vision), and Pdx1 (a gift from Christopher V. Wright (Vanderbilt University, Nashville)). The secondary antibodies were conjugated to rodamin (1∶200, Jackson) and Alexa 488 (1∶200, Molecular Probes). The slides were examined with a Nikon ECLIPSC TE300 and images were obtained with a LEICA DFC400 camera.

### Measurement of islet area

ß-cell mass was measured using Image J software (NIH). Islet numbers and areas were averaged from 6 animals, with six sections from each animal, 250 µm apart.

### RNA extraction and quantitative RT-PCR

Total RNA was prepared from isolated tissues using the RNeasy mini kit (Qiagen) with a modified protocol to purify total RNA containing miRNA from animal tissues. Differential gene expression was confirmed using the SYBR Premix Ex Taq (TAKARA) in accord with the manufacturer's instructions. The reaction was performed using the SYBER Green program on an ABI PRISM 7700 sequence detector system (Applied Biosystems). The expression of mRNA was normalized to that of GAPDH mRNA.

Primer sequences were as follows, ActRIIA: 5′-AGCGGAGCTGACAGTGATTT-3′, 5′-CATACACGCACAACACACCA-3′ ActRIIB: 5′-TGGACATCCATGAGGTGAGA-3′, 5′-CAGCAGCTGTAGTGGCTTCA-3′

### miRNA microarray

Small RNAs were labeled with a miRNA labeling Reagent & Hybridization Kit (Agilent) based on the manufacturer's instructions. The Cy3-labeled RNA molecules were hybridized with a Mouse miRNA microarray (Agilent), consisting of control probes, mismatch probes, and 567 capture probes as registered and annotated in Sanger miRBase v10.1. A DNA MicroArray Scanner (Agilent) was used to scan images. The scanned images were analyzed with Agilent Feature Extractin Ver.9.5.3 (Agilent). Data were normalized globally per array. The net intensity values were normalized to per-chip median values.

### Glucose tolerance test and insulin concentration

After overnight fasting, 2 mg/g (body weight) of glucose was administered intraperitoneally. Blood samples were drawn intraperitoneally from the tail at different times, and the blood glucose concentration was measured with an automatic blood glucose meter, Freestyle Freedom (NIPRO). Whole blood was collected and centrifuged, and the plasma was stored at −80°C. The insulin concentration was measured with an insulin measurement kit (Morinaga) in accordance with the manufacturer's instructions.

### Growth hormone measurements

Growth hormone concentrations of wild-type and *Dicer1*-hypomorphic mice were measured with a rat/mouse growth hormone ELISA kit (LINCO Research).

### Data analysis

Data were analyzed by the one-sample t-test and independent samples t-test. Data are the means±S.D.
